# SOCIODEMOGRAPHIC CHARACTERISTICS RELATED TO KNOWING THE BENEFITS OF BREASTFEEDING

**DOI:** 10.1590/1984-0462/2021/39/2020101

**Published:** 2021-04-02

**Authors:** Viviane Garcia da Silva Alves, Maria Carliana Mota, Carla Pagliari

**Affiliations:** a Instituto de Assistência Médica ao Servidor Público Estadual, São Paulo, SP, Brazil.; b Universidade Federal de Uberlândia, Uberlândia, MG, Brazil.

**Keywords:** Breastfeeding, Milk, human, Postpartum period, Infant, Weaning, Aleitamento materno, Leite materno, Período pós-parto, Lactente, Desmame

## Abstract

**Objective:**

To describe the characteristics of women according to the reported number of benefits of breastfeeding and to verify its association with the duration of this practice until the sixth month of the child’s life.

**Methods:**

This was a qualitative and prospective observational study performed with postpartum mothers in two stages (n=78, and after six months n=62). Generalized linear models were used to identify the profile of the mothers as well as to determine the factors associated with the duration of breastfeeding until the sixth month of the child’s life.

**Results:**

The profile of women who reported fewer benefits (≤3) was: younger age (p=0.008), with lower schooling (p<0.001), single (p=0.02), unemployed (p=0.04) and who attended prenatal care at the public health service (p=0.01). The analysis of the interaction of these factors indicated that women who had only completed elementary school and who attended prenatal care at the public health service (p<0.001) or privately (p=0.01) reported fewer benefits. Factors such as: level of education, marital status, previous education/training about breastfeeding, place of prenatal care and the reported number of benefits were not associated with the duration of breastfeeding until the sixth month of the child’s life.

**Conclusions:**

The lowest number of breastfeeding benefits was reported by women with elementary education and who undewent prenatal care in the public health system or privately. The number of reported benefits was not associated with the duration of this practice until the age of sixth months of the child.

## RESUMO

**Objetivo:**

Descrever o perfil das mulheres de acordo com o número relatado de benefícios do aleitamento materno e verificar sua associação com a duração dessa prática até o 6º mês da criança.

**Métodos:**

Trata-se de um estudo observacional qualitativo e prospectivo realizado com puérperas em duas etapas (n=78 e, após seis meses, n=62). Modelos lineares generalizados foram usados para identificar o perfil das puérperas, assim como para determinar os fatores associados à duração do aleitamento materno até o 6º mês da criança.

**Resultados:**

O perfil das mulheres que relataram menos benefícios (≤3) foi: mulheres mais jovens (p=0,008), com menor nível de escolaridade (p<0,001), solteiras (p=0,02), desempregadas (p=0,04) e que fizeram o pré-natal na rede pública de saúde (p=0,01). A análise da interação desses fatores indicou que as mulheres que tinham somente o ensino fundamental que fizeram pré-natal na rede pública de saúde (p<0,001) ou de modo privado (p=0,01) relataram um número menor de benefícios. Fatores como nível de escolaridade, estado civil, recebimento de prévias orientações sobre o aleitamento materno, local de pré-natal e número de benefícios relatados não se associaram à duração do aleitamento materno até o 6º mês da criança.

**Conclusões:**

O menor número de benefícios do aleitamento materno foi relatado pelas mulheres com ensino fundamental e que fizeram o pré-natal na rede pública de saúde ou de modo privado. O número de benefícios relatados não se associou com a duração dessa prática até o 6º mês de vida.

## INTRODUCTION

The World Health Organization^1^ recommends that breastfeeding be exclusive in the first six months of life. After that period, it is important to introduce other foods and continue breastfeeding until the age of two years or more. However, data from the United Nations International Children’s Emergency Fund - UNICEF) show that, around the world, only 44% of the children aged between 0 and 5 months are exclusively breastfed.^2^

Breastfeeding is essential both for the health of the child and the mother. Besides, it provides several economic and environmental benefits that are acquired in the short and long term.^3^ ^,^ ^4^ Therefore, this practice has a positive repercussion on the children who are breastfed, on the women who breastfeed, and on all of the society.^4^ ^,^ ^5^

When considering the benefits of breastfeeding globally, it is important to highlight the reduction in morbidity and mortality rates. Around the world, it is estimated that the absence of breastfeeding causes, per year: the death of 595,379 children aged from 6 to 59 months due to diarrhea and pneumonia; 974,956 cases of childhood obesity; and death of 98,243 women due to breast cancer, ovarian cancer and type 2 diabetes. These factors could contribute, in a global level, to save 1.1 billion dollars a year.^5^

Breastfeeding is considered a complex phenomenon in which several factors are involved, whose origin can be social, physical, or even psychological. Some examples are: the increasing insertion of women in the labor market, difficulties related to the act of breastfeeding, relationships between the nursing mother, her partner and the family, cultural influences, among many other conditioning factors.^6^ It is important to mention that the knowledge about breastfeeding is considered as a crucial factor, being easily changeable, and able to influence its prevalence.^7^

Even though breastfeeding is part of the public policies of health promotion, it is still necessary to investigate the multiple factors involving the complex act of breastfeeding, also regarding the perception of puerperal women about the advantages of the act. Therefore, the objective of this study was to describe the profile of women according to the reported number of benefits of breastfeeding, and to verify its association with the duration of this practice until the 6^th^ month of the child’s life. The hypotheses of this study were that women with low schooling would present less knowledge about the benefits of breastfeeding, and that higher knowledge would be associated with longer duration of this practice.

## METHOD

The study was approved by the Research Ethics Committee of Instituto de Assistência Médica ao Servidor Público Estadual, protocols n. 2,647,053 and n. 2,953,532, according to the recommendations of the National Health Council, Resolution n 466/2012.

This is a qualitative, prospective, observational study carried out with puerperal women who attended two Basic Health Units (UBS) of a city to the South of Minas Gerais, Brazil. The sample was selected by convenience in May and June, 2018. All puerperal women who searched for care in the referred UBSs in this period were invited to participate in the study, and all of them accepted. The participants were informed about the objective, the methodology and the possibility of no longer participating at any moment. The permission was formalized by the signature of the informed consent form.

The first study phase included 78 puerperal women, with the following inclusion criteria: women in the first 15 postpartum days, aged more than 18 years, who breastfed their children exclusively, predominantly or partially. The second phase took place six months later, when 62 of them were followed-up by telephone.

Two semi-structured interviews were carried out, which allowed the mothers to talk about the subject, and the respective reports were literally transcribed. After this stage, the answers were categorized by semantic approximation, based on content analysis.^8^

The first phase analyzed: sociodemographic aspects (maternal age, number of children, schooling, marital status and occupation); previous gestational factors (number of pregnancies and previous breastfeeding history); factors of the last pregnancy (prenatal location, orientation about breastfeeding, type of delivery, gestational age at birth and child’s age); factors related to breastfeeding (breastfeeding in the first hour, intake of other milks and difficulties to breastfeed); investigation of the benefits of breastfeeding (for the children, the mothers and the family).

After six months, a second interview analyzed: maternal occupational status, dietary habits of the children and continuity of breastfeeding or weaning. The duration of breastfeeding was calculated in number of days, and was obtained using the date of birth and the date of weaning; in case the mother was still breastfeeding, we used the date of birth and the date when the child completed 6 months of life.

Statistical analyses were performed using the Statistical Package for the Social Sciences (SPSS), version 20,0 (SPSS Inc., Chicago, IL, USA), and the analyses that showed p<0.05 were considered statistically significant. The Kolmogorov-Smirnov test was used to determine the normality of the data.

Initially, we calculated the median of the number of reported benefits of breastfeeding, and this number was used to classify the participants in two groups: reported benefits≤median, and reported benefits>median. This classification was used for descriptive analyses and in generalized linear models (GLM).

The categorical variables were presented in frequency and percentage rates, and continuous variables were shown in means and standard deviation, or median and interquartile range. The Pearson chi-square test was used to compare proportion variables. The Student’s t- test or the Mann-Whitney test were used to compare continuous variables in independent samples.

The Spearman correlation test was used to verify a possible correlation between the number of breastfeeding days and the number of reported benefits. This analysis was adjusted for age and maternal occupational status.

The GLMs were adjusted for maternal age, and used to analyze the effects of schooling, marital status, occupational status, previous orientations about the importance of breastfeeding and prenatal location (independent variables) on the number of reported benefits of breastfeeding (dependent variables). Individual tests were performed for each independent variable, and we also assessed the interaction between the investigated factors using the gamma distribution. The GLMs were also used to analyze the effect of schooling, marital status, previous orientations and number of reported benefits of breastfeeding (independent variables) on the duration of breastfeeding until the 6^th^ month of the child’s life (dependent variable). The paired comparison analysis was performed using the sequential Sidak’s test.

## RESULTS

By grouping all of the benefits mentioned by puerperal women, the results show a median of three benefits [3-5]. Table 1 presents demographic, gestational and breastfeeding factors according to the number of reported benefits.

**Table 1:** Sociodemographic and gestational characteristics and factors related to breastfeeding according to the number of reported benefits.

	All n=78	Reported benefits >3 n=36	Reported benefits ≤3 n=42	p-value*
Demographic factors				
Maternal age (years)	28.7±6	30.7±5.0	27.1±6.4	0.008
Number of children	2 [1-2]	2 [1-2]	2 [1-2]	0.22
Schooling				
Elemntary school (%)	23 (29.5)	2 (8.7)	21 (91.3)	<0.001
High school (%)	36 (46.2)	20 (55.6)	16 (44.4)	
Higher Education (%)	19 (24.3)	14 (73.7)	5 (26.3)	
Marital status				
Single (%)	20 (25.6)	4 (20.0)	16 (80.0)	0.02
Married (%)	58 (74.4)	32 (55.0)	26 (45.0)	
Occupational status				
Unemployed (%)	17 (21.8)	5 (29.4)	12 (70.6)	0.04
Student (%)	3 (3.8)	0 (0)	3 (100.0)	
Employed (%)	58 (74.4)	31 (53.4)	27 (45.6)	
Previous gestational factors				
Number of pregnancies	2 [1-2]	2 [1-3]	2 [1-2]	0.12
Has breastfed before?^≠^				
Yes	42 (89.4)	22 (52.4)	20 (47.6)	0.29
No	5 (10.6)	3 (60.0)	2 (40.0)	
Factors of the last pregnancy				
Prenatal location				
Public health network (%)	26 (33.3)	7 (27.0)	19 (73.0)	0.01
Health insurance (%)	37 (47.4)	23 (62.0)	14 (38.0)	
Private (%)	15 (19.2)	6 (40.0)	9 (60.0)	
Previous guidance about the importance of breastfeeding				
Yes	61 (78.2)	26 (43.6)	35 (57.4)	0.23
No	17 (21.8)	10 (58.8)	7 (41.2)	
Type of delivery				
C-section	59 (75.6)	30 (50.8)	29 (49.2)	0.14
Natural	19 (24.4)	6 (31.6)	13 (68.4)	
GA at birth				
Term	76 (97.5)	34 (44.7)	42 (55.3)	0.12
Preterm	2 (2.5)	2 (100.0)	0 (0)	
Child’s age (days)	4.0 [3.7-6.0]	4.0 [3.2-6.0]	4.0 [3.7-7.0]	0.23
Factors of breastfeeding				
Child breastfed in the first hour				
Yes	61 (78.0)	29 (47.5)	32 (52.5)	0.64
No	17 (22.0)	7 (41.2)	10 (58.8)	
Had another type of milk in the 24h prior to the interview				
Yes	13 (16.7)	5 (38.5)	8 (61.5)	0.54
No	65 (83.3)	31 (47.7)	34 (52.3)	
Had difficulties breastfeeding?				
Yes	26 (33.3)	15 (57.7)	11 (42.3)	0.14
No	52 (66.7)	21 (40.4)	31 (59.6)	

Amounts presented as mean±standard deviation if normal distribution, or as median [interquartile range] if not normal, or in number (%) if categorical. ^≠^n=47. GA: gestational age.

Maternal age ranged between 18 and 42 years, and, among the women who reported fewer benefits of breastfeeding, the following characteristics can be mentioned: younger women (p=0.008), with lower schooling (elementary school, p<0.001), single (p=0.02), unemployed (p=0.04), who underwent prenatal care in the public health system (p=0.01). The other variables did not present significant differences between the groups, when compared (Table 1).

The benefits of breastfeeding for children were the most reported ones and presented median of 2 [1-2]. As to the benefits for the mothers who breastfeed, the median was 1 [0-2]. Regarding the family, reports corresponded to 0 [0-1], and 53.8% of the interviewees could not mention any benefit in this category (Chart 1).

**Chart 1:** Categorization of the benefits of breastfeeding reported by mothers.

Reported benefits for the children who are breastfed	n (%)
Immunity	54 (69.2)
Importance for health	22 (28.2)
Nutritional aspects of MM	22 (28.2)
Development	13 (16.7)
Growth	12 (15.4)
MM’s superiority when compared to baby formula	5 (6.4)
Protection of the intestine, preventing colic	4 (5.1)
Loving relationship between mother and child	3 (3.8)
For intelligence	2 (2.6)
For dentition	2 (2.6)
The child is calmer	2 (2.6)
Good for face muscles	1 (1.3)
Knew how to report at least one benefit	74 (94.9)
Could not report any benefit	4 (5.1)
**Benefits reported for the mothers who breastfeed**	**n (%)**
Losing weight and returning to pre-gestational weight	31 (39.7)
Uterine involution	13 (16.7)
Satisfaction, pleasure, happiness to breastfeed	11 (14.1)
Loving relationship between mother and child	18 (23.1)
Prevention of breast cancer	4 (5.1)
Ease, practicality	4 (5.1)
Prevents engorged breasts and breast pain	2 (2.6)
Prevents hemorrhage	1 (1.3)
Contraceptive method	1 (1.3)
Exclusivity of the mother	1 (1.3)
Knew how to report at least one benefit	51 (65.4)
Could not report any benefit	27 (34.6)
**Benefits reported for the family**	n (%)
Financial savings	22 (28.2)
Loving relationship between family members during the act of breastfeeding	10 (12.8)
Healthy child because of breastfeeding, less work for the family and fewer visits to the doctor	6 (7.7)
Ease and practicality	3 (3.8)
The child who breastfeeds is calmer, and that causes less stress to the family	1 (1.3)
Knew how to report at least one benefit	36 (46.2)
Could not report any benefit	42 (53.8)

*The mother could mention more than one benefit of breastfeeding in each category. MM: maternal milk.

When the factors were assessed in an isolated manner, we identified that women with higher education or high school (p<0.001), or those who underwent prenatal care with a private insurance (p=0.04) reported more benefits when compared to women with elementary school and those who underwent prenatal care in the public network, respectively (Table 2). However, after the analysis of interaction between the schooling and prenatal location, it was observed that women who had only completed elementary school and who underwent prenatal care in the public health network (p<0.001) or with private insurance (p=0.01) reported fewer benefits when compared to the women with higher education who underwent prenatal care with private insurance (Table 2).

**Table 2:** Factors associated with the reported benefits (n=78).

Adjusted model^≠^	Mean±SE
Schooling	
Higher education	4.2±0.4ª
High school	3.9±0.2ª
Elementary school	2.5±0.2^b^
Marital status	
Married	3.8±0.2
Single	3.2±0.3
Occupational status	
Employed	3.8±0.3
Unemployed	3.0±0.3
Previous orientation about breastfeeding	
Yes	3.6±0.2
No	3.9±0.4
Prenatal location	
Public health network (%)	3.1±0.3ª
Health insurance (%)	3.8±0.4^ab^
Private (%)	3.9±0.3^b^
**Model of interaction^≠^**	**Mean±SE**
Prenatal location × schooling	
Public health network × elementary school	2.6±0.3^a^
Public health network × high school	3.6±0.4ª^b^
Health insurance × high school	3.5±0.6ª^b^
Health insurance × higher education	3.9±0.6^ab^
Private × elementary school	2.4±0.4^a^
Private × high school	4.1±0.4^ab^
Private × higher education	4.3±0.5^b^

^≠^Generalized linear models adjusted for maternal age and used to analyze the effect of schooling, marital status, occupational status, previous orientation about breastfeeding and prenatal location in relation to the number of reported benefits of breastfeeding. Equal letters indicate that the means do not present statistical differences. SE: standard error.

The results of the interview in the second phase are presented in Table 3. In this stage, the median age of children was 192 days [188-203] (p=0.14), and we observed prevalence of 30.6% of weaning. The children’s age, prevalence of weaning and period of introduction to foods did not present significant differences, according to the number of reported benefits (Table 3). The data also showed that, considering all mothers and after adjustments of age and occupational status, there was no significant correlation between the total days of breastfeeding and number of reported benefits (r=0.18; p=0.16).

**Table 3:** Child’s age, weaning and introduction of other foods obtained in the second interview (n=62).

	All n=62	Reported benefits >3 n=32	Reported benefits ≤3 n=30	p-value*
Child’s age (days)	192 [188-203]	190 [187-204]	197 [189-203]	0.14
Weaning (%)				
- Yes	19 (30.6)	9 (47.4)	10 (52.6)	0.65
- No	43 (69.4)	23 (53.5)	20 (46.5)	
Introduction of foods (months)	5 [5-6]	5 [5-6]	5 [5-6]	0.90

*Amounts in median [interquartile range] for data with non-normal distribution.

Schooling, marital status, previous orientation about breastfeeding, prenatal location and number of reported benefits were not associated with the duration of breastfeeding (Table 4).

**Table 4:** Factors associated with the duration of breastfeeding (n=62).

	Adjusted model^≠^
	Duration of breastfeeding (days)^∞^ Mean±SE
Schooling	
Higher education	140.4±17.7
High school	156.1±15.6
Elementary school	140.4±17.7
Marital status	
Married	152.4±10.4
Single	141.7±37.7
Previous orientation about breastfeeding	
Yes	155.3±11.7
No	139.7±19.9
Prenatal location	
Public health network	175.9±24.5
Health insurance	146.6±14.2
Private	131.6±14.2
Number of reported benefits	
>3	159.8±14.9
≤3	143.2±13.8

^≠^Generalized linear models adjusted for maternal age and occupational status were used to analyze the effect of schooling, marital status, previous orientation and number of reported benefits of breastfeeding in relation to duration of breastfeeding until the 6^th^ month of life. No analyzed factor was significantly associated with the duration of breastfeeding; ^∞^the duration of breastfeeding was obtained considering the child’s date of birth and the date of weaning. In case the mother was still breastfeeding, we used the child’s date of birth and the date when the child completed six months of life. SE: standard error.

## DISCUSSION

This study showed that women with lower schooling (elementary school) who underwent prenatal care in the public health network or with a private insurance reported fewer benefits of breastfeeding. A previous analysis performed in Family Health Units in the South of Brazil assessed the proportion of pregnant women with knowledge about the ideal recommended duration of exclusive breastfeeding.^9^ The results of the mentioned study pointed out that 71.6% of the women (n=151) answered this question correctly, and the highest level of hits occurred among women with higher schooling. Therefore, the authors suggested the need for Family Health Strategy staff to intensify the health education actions about breastfeeding, especially addressing pregnant women with low schooling. A study^10^ conducted in Spain also identified that schooling influenced the knowledge about the theme, and the suggestion was that higher schooling allows mothers to analyze the benefits of breastfeeding with higher level of awareness.^11^

Concerning prenatal locations, health professionals should promote breastfeeding practices in order to reach women in all schooling levels, and this fact was not observed in this study. In this sense, some researchers^12^ emphasize that the orientations received in prenatal care are factors that contribute with the success of breastfeeding, but there should also be an effort in the postpartum period. Other studies^13^ ^,^ ^14^ also point to the need for specific and periodical training for health professionals about the subject, so they can assist in public policies in the health institutions.

The actions of promotion, protection and support to breastfeeding are related to the reduction of childhood mortality,^15^ and the benefits of this practice for the child are the reasons that mostly influence the mother towards the act of breastfeeding.^16^ In this study, the benefits of breastfeeding for infants were the most mentioned ones, and this fact is similar to that of another study,^17^ carried out in Rio Grande do Sul, which showed that 88% (n=35) of puerperal women mentioned growth, and 75% (n=30), immunity and bonding. Besides, the aspects related to the immunity of breastfed children were reported by 69.2% of the interviewees, and this data is similar to that of a study performed in the outpatient clinic of Universidade Federal de Minas Gerais, Belo Horizonte, in which the infants’ protection against diseases was the most mentioned item by participants, when asked about what they considered important in the act of breastfeeding.^18^ When considered altogether, these results suggest that actions that promote breastfeeding should emphasize the benefits affecting the breastfed children. Such an aspect could increase maternal motivation, and, consequently, cause the prevalence of this practice to increase.

As to the benefits of breastfeeding for the mothers who do it, 39.7% of the participants mentioned losing weight and recovering pre-gestational weight. In other studies,^17^ ^,^ ^19^ ^,^ ^20^ these factors were also the most reported ones. In this study, 65.4% of the puerperal women reported at least one benefit for the nursing mother, and this frequency is lower than the results presented in another study carried out with pregnant women assisted in health units in a city of Bahia, from October 2010 to May 2011, in which 88% of the interviewees recognized the importance of breastfeeding for the health of the women.^21^ Therefore, the results identified here show that policies that promote breastfeeding also need to focus on the reflection and dissemination of the several benefits provided to the nursing mother. In fact, breastfeeding helps to recover pre-gestational weight,^22^ however, the benefits go beyond that and can contribute with uterine involution,^23^ prevention of anemia^24^ and reduction in the incidence of chronic conditions, such as diabetes mellitus (types 1 and 2), obesity,^3^ hypertension,^3^ ^,^ ^25^ heart disease, hyperlipidemia and some types of cancer, such as breast and ovarian cancer.^3^

Most of the interviewed puerperal women could not report any benefit of breastfeeding for the family, and 28.2% mentioned aspects related to financial savings. In fact, a global research^5^ on the cost of the absence of breastfeeding estimates that the supply of baby formula in the first two years of life encumbers a family’s wage, in average, in more than 6.1%; when considering low and middle income families, this amount would be even higher. Therefore, it is emphasized that the involvement and participation of family members in breastfeeding is are extremely important. These individuals should be stimulated to participate in health actions developed for pregnant and nursing women, and also be encouraged to provide the support these women need.

In this study, most women breastfed their children until the 6^th^ month of life, and this frequency is similar to that found in another Brazilian study with mothers of preterm newborns, performed by researchers in the city of Vitória, ES, which identified prevalence of breastfeeding of 65.4%.^19^ Such findings also approach a national Brazilian study^26^ which showed that 62.3% of the children in this age group were breastfed. Still considering the Brazilian scenario, there was an increasing tendency between the years of 1986 and 2006. However, after this period, between 2006 and 2013, these indicators remained relatively stable, with 36.6% exclusive breastfeeding in children aged less than 66 months; 45.4% of continued breastfeeding in the 1^st^ year of the child; and 52.1% of breastfeeding in children aged less than 2 years.^27^ These findings reinforce the fact that more mothers should be contemplated with actions of awareness and motivation of this practice, and also point to the need to review the Brazilian public policies, so that new strategies can be used.

The number of reported benefits of breastfeeding was not associated with the number of days of this practice. However, the low total number of benefits of breastfeeding reported by puerperal women may have compromised a possible association between this factor and the duration of breastfeeding. In this sense, Suárez-Cotelo *et al.* ^10^ defend that knowing about the theme influences the exclusivity of this pratice; however, they highlight that this relationship becomes weakened with time. The referred authors also stand up for the importance of following-up these puerperal women, especially in the first three postpartum months, in order to identify the faced difficulties and develop the necessary interventions.

In this study, the fact of receiving previous guidance about breastfeeding was not associated with duration. A study^28^ carried out in Mexico did not identify increment in knowledge about the benefits of breastfeeding even when the women were advised about this practice. However, other authors^10^ defend that women have the right to know the advantages of breastfeeding, once this factor conditions the intention of breastfeeding. These authors also point to the importance for professionals to identify the women with low level of knowledge and develop educational strategies about the subject. Therefore, maternal and child health would benefit from it, and the prevalence of breastfeeding would increase.^7^

This study did not investigate family income, and that can be considered as a limiting factor, since some authors^29^ report the existence of a positive association between income and knowledge about breastfeeding. It is possible to mention other limitations, such as the absence of a sampling calculation, the small number of participants in a convenience sample, the impossibility to assess the quality of the orientations provided by health professionals, and the fact that the study was performed in a city in the countryside of Brazil, which makes it difficult to extrapolate the results to other populations.

Considering the presented results, the conclusion is that puerperal women with lower schooling, who underwent prenatal care in the public health network or with a private insurance, reported fewer benefits of breastfeeding. The assessed factors (schooling, marital status, receiving previous orientation about the theme, prenatal location and number of reported benefits) were not associated with the duration of this practice until the 6^th^ month of the child’s life.
